# Like a jar of flies? A study of self-control in an organizational social dilemma with large stakes

**DOI:** 10.1371/journal.pone.0207808

**Published:** 2018-12-19

**Authors:** Matthew W. McCarter, Jonathan R. Clark, Darcy Fudge Kamal, Abel M. Winn

**Affiliations:** 1 Department of Management, University of Texas–San Antonio, San Antonio, Texas, United States of America; 2 Argyros School of Business & Economics, Chapman University, Orange, California, United States of America; Middlesex University, UNITED KINGDOM

## Abstract

We study the practice of self-control in an organizational social dilemma when the stakes are large, using 47 years of vital census data from 18^th^ century Sweden. From 1750 to 1800, eighty percent of Sweden lived in a simple-structure organization called a *bytvång* or village commons. The amount of resources a village family received was a function of their size. During this period, crop failures left the population facing starvation. Using autoregressive time-series modeling, we test whether the people of Sweden continued to take steps toward increasing the stress on the commons by marrying and birthing children or practiced self-control. We find evidence that the peasantry–with little education, archaic agricultural practices, strong barriers to abortion and infanticide, and pressures by the Church and State to procreate–were less likely to marry and birth children (in or outside of wedlock) when the quality of the previous year’s harvest was poor compared to when it was bounteous. Post hoc analyses support the idea that the reason behind declining fertility after a famine was human decision rather than human physiology. Our findings are consistent with the idea that human population growth is not a social dilemma called a collective trap–which has been the assumption for 50 years. Rather, human population growth may be an individual dilemma–suggesting that members of simple-structured organizations can unilaterally exercise self-control and manage resources through self-organizing.

## Introduction

The practice of self-control is central for the continual functioning of communities, organizations, and society. It is therefore expected that self-control is the topic of conversation among scholars across a breadth of fields that study cooperation; e.g. anthropology [1], management [2], psychology [3], economics [4], sociology [5], political science [6], and human ecology [7]. A literature bridging these fields on self-control and cooperation is the social dilemma paradigm.

The social dilemma paradigm is used to model the tension between self-control and cooperation in public goods dilemmas [8, 9, 10], volunteer dilemmas [11, 12], anti-commons resource dilemmas [13, 14], give-and-take-some dilemmas [15, 16], and commons resource dilemmas [17, 18]. It is the ubiquitous commons resource dilemma that is the focus of the current research. In a commons resource dilemma, individuals are tempted to consume or use a shared resource for personal gain in the short run but in doing so place greater stress on a shared scarce resource in the long run [19]. To avoid the commons’ collapse, individuals must practice self-control–voluntarily [17] or coercively [20].

While the social dilemma paradigm is a popular lens for modeling many real-world commons resource problems about self-control and cooperation, Kerr [21] observes that there are few real-world studies of such. And, the few field studies we do have make it challenging to test competing theories because of the data constraints necessary [22, 23]. Of course, the control of the behavioral laboratory provides researchers of social dilemmas excellent opportunity to test competing theories with voluminous amounts of insight about self-control and cooperation [24, 25, 26]. For decades, however, social dilemma researchers have issued calls for field studies to examine the relationship between self-control and cooperation [27]. One of the reasons for these calls is the limitation of generalizability: laboratory subjects are typically volunteers who do not represent the general population and the stakes involved in laboratory commons resource dilemmas are small compared to stakes in the real-world problems they are used to model [28].

In the current research, we study self-control and cooperation of a population in a social dilemma: 18^th^ century Swedes in their native land. The social dilemma is a village commons called the *bytvång*, where food and fuel was produced collectively and the amount of commons resources (e.g. food and fuel) a family received was a function of family size [29]. As we detail below, the village commoners–who constituted 80% of the nation’s population [30]–had little education [31], used archaic agricultural practices [32], faced strong barriers to abortion and infanticide [33], and were pressured by the Church and State to procreate [34]. The issues of self-control and cooperation here are that each household had incentive (and few barriers) to have as many children as possible but each additional child adds a more stress on the village commons. These conditions constitute an attractive setting for testing two competing predictions about self-control and population growth. The *externality approach* submits that population growth is a type of social dilemma called a collective trap, predicting that people, living in a society where resources are shared, will not restrain reproduction unless increased bureaucracy through centralized control is employed to avoid overpopulation and starvation [7, 22]. The *internality approach* posits that population growth is an individual trap, suggesting that people can unilaterally exercise self-control and manage population growth through self-organizing [35, 23].

## Theoretical background and propositions

### The social dilemma paradigm in the conversation of self-control, cooperation, and human population growth

*Social dilemmas* are interdependent situations where individually rational behavior in the short term leads to collective ruin in the long term and include public goods provision and volunteering and helping behaviors [3]. Under the social dilemma umbrella rests a set of social decision problems that we are interested in here called social traps. *Social traps* are circumstances where individual behaviors in the short run bring a person benefits, while creating negative consequences for them in the long run [36, 5].

As reviewed in Messick and Brewer’s [19] seminal paper, there are two types of social traps. The first type is an *individual trap*, where a person is tempted to indulge in something in the short run but must incur the consequences in the long run [37]. For an unwise doctoral student, the enjoyment of playing nonstop Plants versus Zombies® is immediate, the stress of going on the job market with no journal publications and a weak dissertation comes later.

The second type is a collective trap. *Collective traps* are like individual traps, the only difference being that, when individuals enjoy benefits in the short run, the consequences are incurred by others in the long run. Further, the long-term consequences grow as more individuals enjoy the short-term benefits. There are daily benefits for a director passing administrative work to a common department secretary, but if every director in the department does this, then the long-term consequences include a burned out, ornery secretary who is looking for another job. Each social trap is the metaphor used by the competing approaches to human population growth. Table 1 summarizes each approach’s key papers, field sources, assumptions, and predictions.

**Table 1:** A summary and comparison of the two approaches to the social dilemma of self-control, cooperation, and human population growth.

* *	Externality Approach	Internality Approach
*Field*	Human ecology, political science	Business economics, political science
*Foundational papers*	Malthus [39], Hardin [7], Ehrlich [40]	Godwin [47], Simon [23, 35]
*Key assumptions*	• Humans are like many animals: self-interested, absent of foresight, and unable to exercise self-control without coercion.	• Humans are unique from most animals: while self-interested, they have foresight, aware of their natural environment, and exercise self-control without coercion.
	• Children-rearing costs are less for the family than the community.	• Children-rearing costs are more for the family than the community.
*Key proposition*	Population growth is a collective trap where a person must decide between enjoying a short-term benefit while passing along a collective cost in the long run.	Population growth is an individual trap where a person must decide between enjoying a short-term benefit while racking up a private cost in the long run.

### The externality approach to human population growth

Equating population growth to a collective trap is what we term the *externality approach*. The externality approach to human population growth is old, going at least as far back as the third-century Christian writer Tertullian [38], who said the following about the population growth:

What most frequently meets our view (and occasions complaint), is our teeming population: our numbers are burdensome to the world, which can hardly supply us from its natural elements; our wants grow more and more keen, and our complaints more bitter in all mouths, whilst Nature fails in affording us her usual sustenance. In very deed, pestilence, and famine, and wars, and earthquakes have to be regarded as a remedy for nations, as the means of pruning the luxuriance of the human race (p. 308).

To the best of our knowledge, the first to detail the “burdensomeness” of population growth was the cleric and mathematician Thomas Malthus [39] in the 18^th^ century, whose arguments were later restored to popularity in the 1960s by the human ecologist Garrett Hardin [7] and the biologist Paul Ehrlich [40].

Hardin [7] elegantly likens population growth to a commons grazing meadow in the seminal, well-cited paper *Tragedy of the Commons*. Consider a meadow owned in common by many shepherds–each with a herd. The shepherds independently decide how many animals to place in the meadow for fattening and later consumption or sale. The benefits of a fatter animal are internalized by the individual shepherd, while the costs of depleting the grass are spread across the entire community of shepherds. Sustainable grazing requires a shepherd to place animals on the commons until the marginal benefit of the next animal is equal to the marginal cost to the collective. But a rationally self-interested shepherd will place animals on the commons until the marginal benefit is equal to *his share* of the marginal collective costs. The trap springs when enough shepherds behave rationally: the meadow is overgrazed, the herds starve, and the value of the meadow is lost.

#### Assumptions of the externality approach

Applying the commons metaphor to population growth, the externality approach assumes two things. First, humans are like many animals: they are slothful, lack foresight, and cannot exercise self-control without coercion. These assumptions about human nature are comparable to the assumptions underlying McGregor’s enunciation of Theory X; the view that people need rules, direct supervision and coercion to keep them in line [41]. The following excerpts illustrate the assumption:

[Humans] seldom take really adequate account of future threats … Most of the principles worked out for populations of nonhuman animals apply with little change to human populations [42, p. 602].

If too many animals are devouring it, the food supply declines; too little food, the supply of animals declines. … *Homo sapiens* are no exception to that rule [43, p. 66].

Most of the abilities of men, however one measures them, are shared with other animals; only a few, like literacy, are confined to the human species [44, p. 162].

The underlying attitude [that population growth is not a collective trap] is pre-Darwinian: it baldly assumes that the laws of nature which govern all other species of plants and animals were negated for man by the God of Genesis. Man is saved by the formula, “X will provide”, where “X” may be God, Providence, or Science [45, p. 182].

The second assumption is that the costs of rearing children are less for the family than the community. Hardin [22] submits that the cost of producing and providing food, water, and protection are spread across many citizens while the benefits of having a child is internalized by the family; e.g. sexual relations, extra monetary resources such as food stamps and subsidized housing and medical care. Because of the asymmetry of costs to benefits, Hardin [7] posits that an individual is incentivized to bear as many children as possible–like a shepherd is incentivized to add as many animals to the commons as possible. However, each additional child is another mouth for a community to feed, clothe, and protect; and each additional child moves the community closer to exhausting the commons [40]. Without “mutual coercion” by a “strong and farsighted sovereign,” Hardin [22] maintains that the tragedy of the commons is inevitable and will consequently bring mass starvation and environmental ruin (p. 564).

#### Externality approach’s proposition

The externality approach maintains that human population growth is curtailed not because humans adapt to anticipated crises, but either because of exogenous shocks like famines, wars, and natural disasters [38] or by centralized authority that controls growth [45]. The animalistic nature of humans coupled with the assumption that the costs of rearing children are greater for the community than the family leads to the following fundamental proposition of the externality approach.

*Proposition 1*_*Externality*_: Population growth is a collective trap where a person must balance a short-term private benefit against a long-term collective cost.

### The internality approach to human population growth

In contrast to the externality approach, the *internality approach* treats human population growth as an individual trap. The internality approach can be likened to a cornucopia–the “horn of plenty” that was broken from the head of Amalthea by the infant Zeus [46]. The horn was said to give to its possessor a limitless source of life, energy, and innovation.

The idea of the human mind as a limitless resource begins at least as far back as the political philosopher William Godwin’s work [47] *Enquiry Concerning Political Justice* when he suggested that human beings are different from animals in that humans can innovate and improve their quality of life. The business economist Julian Simon took Godwin’s idea of the human mind further. Simon [35] submitted that the human mind is the “ultimate resource” on earth and–like a cornucopia–can produce an endless supply of innovation and solutions to societal and environmental problems.

#### Assumptions of the internality approach

Assuming the human mind–unlike other animals’–is significant in its capacity to create value and solve problems, Simon [23] submits that people–even the least educated and most impoverished–have foresight and the ability to exercise self-control when making reproductive decisions. This perspective is reminiscent of the assumptions underlying McGregor’s Theory Y; the view that people are naturally creative, desire to better themselves and improve the social circumstances in which they live and work [41]. Simon’s [23] reasoning for the human mind being “the ultimate resource” stems from his empirical observation that, because of human innovation, human life on Earth has continually improved over the long run: humans are living longer (and more comfortably) on average (be they in developed or developing countries) than they did hundreds and thousands of years ago; the amount of natural resources (all metals and fossil fuels) in reserve have continually increased since their discovery; and the cost of food production and water sanitization (controlling for inflation) has continually decreased on a global scale since records were first kept [35, 23]; Moore and Simon, 2001); all this, despite exponential growth in Earth’s population.

The internality approach also differs from the externality approach when it comes to assuming how costs of childrearing are distributed between the family and society. Simon [23] postulates that most the costs of a child are internal to the family, in the form of money spent on resources as well as time and energy expended in child rearing. More specifically, Simon submits that the costs of rearing a child are internalized and higher for the individual compared to those borne by the community. After a child is born, the parents are typically left with the responsibility to feed, dress, protect, wash, listen to, talk to, nurse, and play with the child for at least two decades. Consequently, the internality approach suggests that because of these characteristics of child-bearing and rearing, individual and collective goals are well aligned when it comes to human population growth. In other words, people will engage in behaviors to increase the population–e.g. entering marriage or bearing children–more often when resources are abundant compared to when it is a time of scarcity [23].

Some empirical evidence supports the idea that humans, who lived before a demographic transition of pre-modern to a post-modern society [48], are quite capable of curtailing reproduction. Kolk’s [49] study of women in 19^th^ century northern Sweden found intentional spacing of births based on a host of family and geographic factors. Bengtsson and Dribe [50] found that Swedish villagers in five southern parishes, restricted child bearing in response to rising grain prices.

#### Internality approach’s proposition

Those relative costs serve to align individual interests with collective interests. Consequently, the internality approach views human population growth as an issue around which society may be best left to self-organize. Self-organization is a process by which individual agents acting with minimal external interference collectively produce some degree of large-scale order [51]. Hayek [51] described such self-organization as a powerful possibility for organizing social systems: “…the only possibility of transcending the capacity of individual minds is to rely on those super-personal ‘self-organizing’ forces which create spontaneous order” (p. 54). Thus, the following proposition runs counter to the externality approach,

*Proposition 2*_*Internality*_: Population growth is an individual trap where a person must balance a short-term benefit against a long-term private cost.

### A long debate and a confusion gap

Debates between proponents of the internality and externality approaches span centuries; e.g. the penned debates between Thomas Malthus and William Godwin in the 18^th^ and 19^th^ centuries [39, 47];, the televised debates among Julian Simon, Garrett Hardin, and Paul Ehrlich in the 1980s [52, 53], and the online debates among academicians, journalists, and leaders of non-government organizations [54]. Despite these debates, Dempsey [55] observes that research on the social dilemma of population has remained “conceptual [and] discussed in general” without empirical assessment (p. 2556). Indeed, we are unaware of any empirical tests motivated by these competing approaches, which is surprising given the sizeable economic, organization, political, and social consequences associated with this issue.

Meaningful attempts to close the “confusion gap” [56] about human population growth have proven elusive because doing so requires a dataset satisfying numerous criteria. The people studied would have to live in a commons welfare system that spread the resource costs across the local population. The people would need to have little or no education, which might otherwise encourage self-control. External coercion could be a substitute for self-control, so, in the ideal dataset, government and religion would have a minimal involvement in the people’s home life and family planning–unless of course they encouraged large families. Technology (for such things as food preservation, crop production, and harvesting) would need to be primitive and stagnant, ensuring that any shocks to the living environment–such as a drought–are caused exogenously by nature. The institutions of abortion and infanticide would need to be taboo and unavailable. Lastly, the society in question would have vital statistics giving us an idea of the people’s health and life quality over a long period. In this study, we rely on an historical population that meets each of these criteria: the bytvång or village compulsion system of 18^th^ century Sweden.

## Empirical context and hypotheses: The village compulsion system in 18^th^ century sweden

### A Commons and simple structure organization

The lives of 18^th^ century Swedes were largely organized–socially, politically, economically–by local institutions, most notably the parish, or church congregation, overseen by a local priest who had responsibility for both ecclesiastical and secular matters. While the priests had some level of authority, Sundin and Willner [57] observe that local issues were largely handled in a participative way through “shared interests, negotiations and compromises” (p. 45). Within this social structure, agriculture was largely a communal enterprise, with farms and fields grouped into common-field villages known as the *“village compulsion system”* or *bytvång*.

#### The bytvång was a tragedy of the commons

The *bytvång* originated in the Middle Ages of Sweden and was dominant until the beginning of 19^th^ century [29]. Thomas [30] summarized the bytvång this way:

The greater part of farming was done on the common-field basis. Farm dwellings were grouped in compact, village-like formations, and individual holdings consisted of many strips of land, often widely separated. Much of the pasture and wooded land was held in common (p. 49). … Animal husbandry … was often a communal enterprise, i.e. one or more persons from the village took care of all the animals (p. 50). … The farm household provided all necessities, and the round of harvesting, plowing, spinning and weaving, milling and baking, brewing and candle-making, forging and joining, was predetermined for the individual farmer by the exigencies of common-field farming and of rural isolation (p. 51).

The assignment of tasks was determined by either a village alderman or council of village families [29]–both of whom were elected by the village body [58]. There was no visible pattern as to what encouraged a village to use an alderman or council. The additional duty of the village leadership was primarily the gathering of village taxes (in the form of produce and animals) for the magistrate’s representative to collect. As Isaksson [59] observed, where disagreements about labor or production arose, the *bytvång* allowed “farmers to meet and have verbal agreements over shared ownership responsibilities” (p. 125).

However, voluntary leadership [60], group meetings [61], and verbal agreements [62] do not guarantee collective action, and this was indeed the case in the bytvång. The 18^th^ century bytvång constituted a commons resource. Hallendorff and Schück [63] describe the tragedy of the commons facing the bytvång in this way:

The homesteads of the different households, grouped closely together, formed the center of such a village community. They were surrounded by cultivated fields, divided up between the villagers, and beyond the fields lay the common grazing land and forests. The arable land belonging to each freeman consisted of several small strips separated one from the other and therefore the farming was done collectively. The whole village ploughed, sowed and reaped at the same time and in all important matters [regarding food production and work] appears to have consulted together and acted as a cooperate body (p. 44).

Heckscher [29] goes further, recording a journalist’s 1755 CE account about the frustrations of “togetherness” with the bytvång.

The sharing of fields, meadows, woods, and wild lands is but the nurse of a country’s poverty. For as long as no one looks after his own, all husbandry is capricious as the moon, though rather on the wane than on the wax. … Where neighbors are sharing fields and pastures, the fields are often not plowed or else they are not plowed in time. One must sow when his neighbor sows, whether the soil is ready or not. One must cut when his neighbor cuts, whether the crop be ripe or not. One must not change his crop to refresh the soil and the corn lest the neighbor protest it. … One must to his great harm, let cattle grace the fields in springtime and fall, as the neighbor may please (p. 27). … Sharing in woods and wild lands is equally if not more harmful. Here everyone follows the old rule, “One shoots and another snatches; everyone keeps what he catches” (p. 29).

#### The bytvång was a simple structure organization

Across Sweden, each parish, and within them, each bytvång, operated as an independent organization. Westley [64] defines an *organization* as “a series of interlocking routines, habituated action patterns that bring the same people together around the same activities in the same time and places” (p. 339). For members of a bytvång, each family had a series of interdependent routines of chores; e.g. strips of land to till and animals to tend and graze. Many of these farming activities brought the members together in regular village meetings to make assignments and reallocate resources as needed [59].

Like contemporary organizations, village leadership was unable to legislate the villagers’ efforts, leaving members of the bytvång could shirk assignments and use resources in excess of the village rationing rules. The relationship of the organization’s inputs to outputs–and to a lesser extent the village’s size–made it difficult for either the alderman or village council to coerce people to work: as with contemporary organizations, it was challenging for management to legislate effort of subordinates because the shirker can appear to be working while not [65]. As recorded by Heckscher [29], it was not uncommon for a person to take a little extra firewood, exert a little less effort in plowing the fields, or graze the animals a bit longer on the meadow than allowed. Consequently, Heckscher [29] described the state of many villages in this way: “it is fairly clear that such communism in many cases led to neglect and decay” (p.29).

The bytvång also met the five conditions of Mintzberg’s [66] simple structure organization. First, coordination of tasks came from either a village alderman or village council [58], who acted as the operating core of the organization when attempting to coordinate chores among individual households. In fact, the records of Heckscher [29] and Isaksson [59] suggest that most communication and coordination among families were informal–what Mintzberg [66] termed “mutual adjustment” (p. 4).

Second, the internal environment of a bytvång and its members was simple: farm the commons, pay taxes, and survive. But the external environment was dynamic, necessitating organizational members to respond to challenges as they arose. The challenges included wars (it was not uncommon during the 18^th^ century for Sweden to be actively fighting in two simultaneously) and harvest failures that left villagers facing potential starvation.

Third, Thomas’ [30] record suggests that while the bytvång had Provincial and local rules when it came to tax regimes and crime (e.g. murder, infanticide, and infidelity), its division of labor was loose: there was little to no job formalization or specialization among members. Rather, the types and breadth of jobs families undertook changed considerably from time to time as a function of family size. Village members were interchangeable in their tasks, and, if the head of a homestead became incapable of performing the duties (because of death, health problems, or being drafted into military service), then the homestead responsibilities passed to the eldest son, allowing for business to carry on as usual.

Fourth, the differentiation among bytvång members was minimal. There was little variance as to what any village produced: barley and rye were the common cereals and cows and pigs were the common animal products [30]. Magnusson’s [32] assessment of pre-industrial agricultural practices in Sweden suggests that differentiation did not occur until well into the 19^th^ century.

Lastly, the managerial hierarchy of the bytvång was minimal. Below the alderman or council were the village families and boarding laborers who worked the same fields, pastures, and forests [29]. Further, there is little evidence from the records of Thomas [30] or Heckscher [29] that the bureaucracy of the bytvång was beyond two levels.

### Additional characteristics of the bytvång

#### Individual villages had considerable discretion–independent of the parish and village council–of how to manage their affairs

Sundin’s [67] record of parish operations in 18^th^ century Sweden provides a detailed picture as to the role of the parish in local villagers’ lives. While the parish spoke with investiture of authority from the King, Sweden’s size and its sparse population made centralized control difficult. The monarchy leaned heavily on local village councils and aldermen to manage farming and collecting taxes and used the local parish to mete out justice when laws were broken by local villagers; e.g. drinking on the Sabbath and not memorizing material for Catechism. Further, the introduction of the religious doctrine of pietism, which Swedish veterans brought with them back from the 100-years war against Russia in the 1720s, placed greater emphasis on teaching the individual “to guide himself (sic.) instead of being guided by rules and punishment from outside” (p. 23).

The village council’s extent of power seemed to also stretch only so far, with its primary function was for allocating chores in the village [58]. Marriage was economically costly for the bride and groom, and therefore marriages were postponed during times of economic hardship [68]. But such choices were left primarily to the marrying parties, and Lundh’s [69] record suggests that a village council’s involvement in postponing matrimonies were restricted to well-to-do, large-land holding families who made up a small percentage of the population.

Overall, Sundin [67] maintains the view that, in 18^th^ century Sweden, the governance of the domestic lives of villagers was left to the parents, who were deemed “masters … teachers and rulers of the domestic sphere” (p. 18). Sundin [67] goes further, observing that the village compulsion system, and social system of Sweden in general, “rested upon the household as the primary unit with rights and duties” (p. 20). Therefore, there is little evidence in the record that fertility and marriages were guided by an invisible or visible hand of the Church or state.

#### The commons property regime was inert

The bytvång was an inert property regime. Despite some attempts to enclose and privatize land, 80% of Swedes (constituting primarily of the peasantry) lived in parishes under the village commons system from the Middle Ages to the 19^th^ century [30]. The peasantry, cottagers, paupers, and crofters represented at least 70% of the total Swedish population, while parish blacksmiths and merchants made up between 10–15%. The residual population was the clergy (who about 5% of the total population), largely engaged as parish priests, and the aristocracy (who made up less than 1% of the population). These demographics remained close to constant during the period we study.

The records of Heckscher [29], Kent [34], and Thomas [30] concur that property reform by the government was “slow” to take effect and met “considerable resistance” from Sweden’s population in the 18^th^ century. Scott [31] described the inertia of the village commons changing to a contemporary privatized system in the 18^th^ century in this way:

For the eighteenth century the reduction [from a village commons to a private property system] was seldom more than from 43 strips [of farmland] to 5 or from 30 strips to 6 … Occasionally one could see lone houses in the fields instead of the cluster of cottages in the villages, but *the real reorganization of rural life did not come in the eighteenth century* (p. 261–262, emphasis added).

#### The agricultural technology was archaic

Sweden’s agricultural methods made little progress during the 18^th^ century. Magnusson [32] records that new technologies–e.g. improved plows and sowing machines–were slow to take effect until well into the 19^th^ century in Sweden:

We must avoid exaggerating here the significance of the optimism about [agricultural] technology. One example was the sowing machine which had already appeared in several designs in the eighteenth century … [but] was hardly used even in large scale farming before the 1850s. … *[T]here was no real breakthrough in agriculture based on the more advanced use of farming implements and machinery until the second half of the nineteenth century* (p. 13, emphasis added).

As the record of Thomas [30] suggests, food could neither be preserved nor stored for any lengthy period, and the Swedish government’s constant engagement in war with neighboring countries made importing food impossible. When the harvest was poor, the remainder of the year was grim for the people.

#### Education was poor and narrow in scope

Most Swedes had little access to education beyond religious instruction. Scott [31] highlights the dearth of formal education in 18^th^ century Sweden:

The Latin Schools from the Middle Ages and the later gymnasia [or preparatory schools] in *the cathedral towns had offered education for a small elite*. In the early nineteenth century only about half of the parishes had elementary schools, and these were for only one-half of the population, the boys. The well-to-do could provide instruction at home by parents or by tutors, but *the mass of rural youth received no education beyond Catechism* (p. 351, emphasis added).

Literacy was common among all classes of Swedes, but it was focused almost exclusively on Catechism. Catechism primarily taught the doctrines of The Church of Sweden, and should a child be destined “to carry on the farm, [then] … no ‘superfluous knowledge’” (p. 351) was deemed necessary [31].

#### Large families were encouraged, while abortion and infanticide were taboo and harshly sanctioned

Through edict and the Church of Sweden, the Swedish monarchy encouraged population growth throughout the country in the 18^th^ century, and discouraged depopulation [34]. The encouragement was due to Sweden coming out of and still being engaged in several wars. Further, from the mid-1600s to the 1800s, the Church of Sweden maintained close tabs on marriages and the birthing of children. Infanticide and abortion were rare events in 18^th^ century Sweden, involving on average 3.8% of the total births from 1750 to 1800 CE, and these events almost exclusively among unwed expecting mothers [33]. When either did occur (or were attempted), severe consequences for the mother and any involved parties ensued.

As detailed by Liljeström [33], the initial institution for dealing with unwed expecting mothers was harsh deterrence. Should neighboring villagers know that a woman was pregnant outside of wedlock, then the village priest would be notified, and a public shaming would occur, placing the woman in physical danger. The mother was then treated as a social outcast and often left alone to fare for herself. Abortion and infanticide was punishable by death, and anyone who participated in the act, or who knew of it but did not report it to the clergy was also put to death.

Sandin [70] reports that from 1750 to 1800 CE, the way society (i.e., the local parish) dealt with unwed expecting mothers changed dramatically, though abortion and infanticide remained taboo and rare. The deterrent approach persisted for centuries, but regime changes in the aristocracy and a doctrinal shift in The Church of Sweden from punishment to repentance changed societal attitudes starting in the 1780s.

Through local parishes, unwed expecting parents were now encouraged to marry, and should they marry before the birth, the child was considered legitimate. If marriage was not possible, adoption for the unborn child was encouraged and arranged. In either instance, the expecting mother was not seen as an outcast but rather seen as someone in need of spiritual, emotional, and social support. This softer approach by the parish priests still placed strong pressures against abortion and infanticide. In fact, Sandin [70] suggests that the softer approach encouraged unwed motherhood, placing further stress on the village commons. The taboo of abortion and infanticide left abstinence and (the less reliable) *coitus interruptus* as the methods for curbing fertility in 18^th^ century Sweden [50].

### Hypotheses

The village commons was prolific, its agricultural practices archaic, the education poor, encouragement for large families strong, and opportunity for infanticide and abortion rare for most 18^th^ century Swedes. It would therefore seem that 18^th^ century Sweden is an attractive setting for testing the explanatory power of the externality and internality approaches to the relationship between self-control and population growth. Should we operationalize the propositions of the externality and internality approaches, then we have the following hypotheses.

*Hypothesis 1*_*Null–Externality*_: There will be no relationship between 18^th^ century Sweden’s harvest and marriage and birthrates in the following and second year, ceteris paribus.

*Hypothesis 2*_*Alternative–Internality*_: There will be a positive relationship between an 18^th^ century Sweden’s harvest and marriage and birthrates in the following and second year, ceteris paribus.

## Method

### Overview of data structure

The data used to examine our competing hypotheses were primarily national data on 18^th^ century Sweden stretching over a sample of 46 years (N = 46). The vital and economic records in Sweden from 1750 to 1933 CE were extracted from a publication compiled by sociologist Dorothy Thomas [30], *Social and Economic Aspects of Swedish Population Movements*. Thomas’ [30] original publication is publicly available in English and includes information on annual harvests, birth rates, death rates (including infant death rates), marriage rates, fertility rates, population, and migration balance, among other variables.

In addition to these data, we relied on two additional sources. First, health economists Sandberg and Steckel [71] published an analysis of Swedish heights as a proxy for the citizen’s health for the 18^th^ century. The data were collected from Swedish “muster rolls” (i.e. measurements of individual male members of the army) located at the War Archives in Stockholm, Sweden. The authors kindly provided us with these annualized height data along with each soldier’s year and province of their birth.

Second, we consulted the historical record for information on Swedish governments, including parliamentary versus monarchical rule and, where applicable, the monarch in power. From these sources, we also derived information on the presence or absence of war. While the information we compiled from the historical record is available from a variety of publicly available sources, one that was particularly useful was historian Cronholm’s [72] book, *A History of Sweden from the Earliest Times to the Present Day*.

Our final data set consists of observations covering the years 1753 through 1800 CE. Each observation in the dataset represents a single Swedish-year.

### Measurement and variables

#### Dependent variables

We examine three dependent variables in our analysis, each extracted from Thomas [30]. The first is marriage rate, the primary gateway to population growth by fertility in 18^th^ century Sweden. The marriage rate (*MR*_t_) is expressed for a given year (t) as the rate of males marrying for the first time per 1,000 males ages 20 to 50. The married fertility rate (*MFR*_t_) is expressed for a given year (t) as the national rate of fertility per 1,000 married females ages 15 to 45 years. The unmarried fertility rate (*UFR*_t_) is expressed for a given year (t) as the rate of fertility per 1,000 unmarried females ages 20 to 45.

#### Independent variables

The main independent variable in our analysis relates to the annual harvest in Sweden. These data are taken from Thomas [30], who recorded the original data from Sundbärg’s [73] government report. Since harvests likely have a lagging effect on behavior, particularly fertility, and those effects may linger, particularly at the extremes, we construct our models to account for the current year’s harvest (*HARV*_t_), the immediate prior year’s harvest (*HARV*_t-1_) and the harvest from two years ago (*HARV*_t-2_). Harvest quality is measured using a nine-point index derived from a report on historical population statistics from 1876. The index is based on estimates from Statskontoret (The Swedish Agency for Public Management), which has responsibility for the analysis of state activities in Sweden. The index’s range extends from 0 (complete famine) up to 9 (peak abundance and quality), with 6 being an average harvest. The translation from Swedish to English concerning the harvest index, as found in Sundbärg [73], is as follows:

The number 6 means approximately average harvest and number 9 abundant. The numbers cover the years 1748–1864 and are obtained from background reports to population statistics of 1876 and refer to estimates from Statskontoret [i.e. the Government authority responsible for organization and evaluation of public sector]. … Naturally this is evidence that the basis of estimates changed as time went by; however, it is likely that year-to-year changes were greater in older times than now [in the late 1800s]. One of the fruits of rational agriculture should be that the farmer is not so dependent on weather conditions now compared to in older times. … [O]ne should be aware that the estimates refer to the total harvest, including cattle feed which is of great importance, and that the estimates refer not only to the quantity of the harvest but also its quality (p. 58–59). (Translated by Dr. Clas Wihlborg of Chapman University on November 11, 2015.)

#### Control variables

We control for several critical socio-economic factors that may relate to fertility rates and harvests or both. Migration balance (*MB*_t_) represents the difference between the number of people who entered the country and the number of people who left the country during the year in question. We control for migratory patterns since it may have a material impact on both productive and reproductive tendencies.

We control for adult height (*HEIGHT*_t_), measured as described previously, as a proxy for the general health and physical strength of the population in a given year [71].

We include total population (*POP*_t_) to capture the effect that aggregate size and a growing (or declining) population may have on productive and reproductive tendencies.

We include the infant death rate (*IDR*_t_), measured as the number of deaths of children aged one year or less per 1,000 live births in a given year. Fluctuations in this indicator may be related to the harvest but, perhaps more importantly, may have cognitive implications for fertility-related decision-making. Samuelsson, Rådestad, and Segesten [74] remind us that parents often experience severe trauma when their infant child dies, making them apprehensive to having more children in the future.

We control, using dummy variables, for the ruling government in power during the year in question, as government policies and practices should be expected to relate to both productive and reproductive tendencies. Over the course of our study period there were six governments that held ruling power in Sweden: The Hats Party (parliamentary rule) (omitted), the Caps Party (parliamentary rule) (Gov*CAPS*_t_), Gustav III of Sweden (monarch) (Gov*GIII*_t_), Regent Duck Charles (monarch) (Gov*DUCK*_t_), Gustav Adolf Reuterholm (monarch) (Gov*GAD*_t_), and Gustav IV Adolf of Sweden (monarch) (Gov*GIV*_t_).

Finally, we include an indicator for the extent to which the country was at war during the year in question (*WAR*_t_). As Sweden was involved in several–sometimes overlapping–wars over the study period, we capture this variable as the number of wars being fought during the year in question. War is, of course, an encompassing experience for individuals, families and nations and, given its potential for varied and far-reaching socio-economic impact, we included this important indicator in our models.

### Analysis

Given the time-series nature of our data, we rely on autoregressive time-series models to allow for autoregressive patterns. More specifically, we model our data using autoregressive- moving average models with exogenous inputs (ARMAX). Autoregressive models predict current values of a dependent variable based on lagged, or past, values of the variable. ARMAX models are a special case of these models that allow for the inclusion of independent covariates, in addition to the autoregressive terms.

One of the key decisions when using autoregressive models concerns the number of autoregressive lags to include. While there are several methods proposed to identify the optimal number of autoregressive lags, including the Ng-Perron Sequential t and the Schwartz Information Criterion (SIC), we rely on the MAIC, as prior literature has suggested that it produces superior results [75]. We use the Dickey-Fuller Generalized Least Squares (DFGLS) procedure in Stata to calculate the MAIC for each of our dependent variables. The DFGLS procedure is similar to an augmented Dickey-Fuller test except that the detrending of the time series data is accomplished by GLS regression. The results suggest that the optimal autoregressive lags for each dependent variable are as follows: (i) for *MFR*_t_, 3 lags (Min MAIC = 6.432675, with RMSE = 16.09338), (ii) for *UFR*_t_, 2 lags (Min MAIC = .9313357 with RMSE = 1.386978), and (iii) for *MR*_t_, 1 lag (Min MAIC = 4.673951 with RMSE = 7.481457). Accordingly, the primary models for each of our dependent variables take the following form:

$${MFR}_{t}=\alpha +\beta_{1}{HARV}_{t}+\beta_{2}{HARV}_{t-1}+\beta_{3}{HARV}_{t-2}+\beta_{4}{MFR}_{t-1}+\beta_{5}{MFR}_{t-2}+\beta_{6}{MFR}_{t-3}+\beta_{7}X_{t}+\varepsilon_{t}$$ (1)

$${UFR}_{t}=\alpha +\beta_{1}{HARV}_{t}+\beta_{2}{HARV}_{t-1}+\beta_{3}{HARV}_{t-2}+\beta_{4}{UFR}_{t-1}+\beta_{5}{UFR}_{t-2}+\beta_{7}X_{t}+\varepsilon_{t}$$ (2)

$${MR}_{t}=\alpha +\beta_{1}{HARV}_{t}+\beta_{2}{HARV}_{t-1}+\beta_{3}{HARV}_{t-2}+\beta_{4}{MR}_{t-1}+\beta_{7}X_{t}+\varepsilon_{t}$$ (3)

Where each of the dependent variables and the harvest variables are as described above, *MFR*_t-1_, *MFR*_t-2_, *MFR*_t-3_, *UFR*_t-1_, *UFR*_t-2_, and *MR*_t-1_ represent the autoregressive lags, and X is a vector of covariates, consisting of the control variables described previously.

## Results

**Fig 1:**
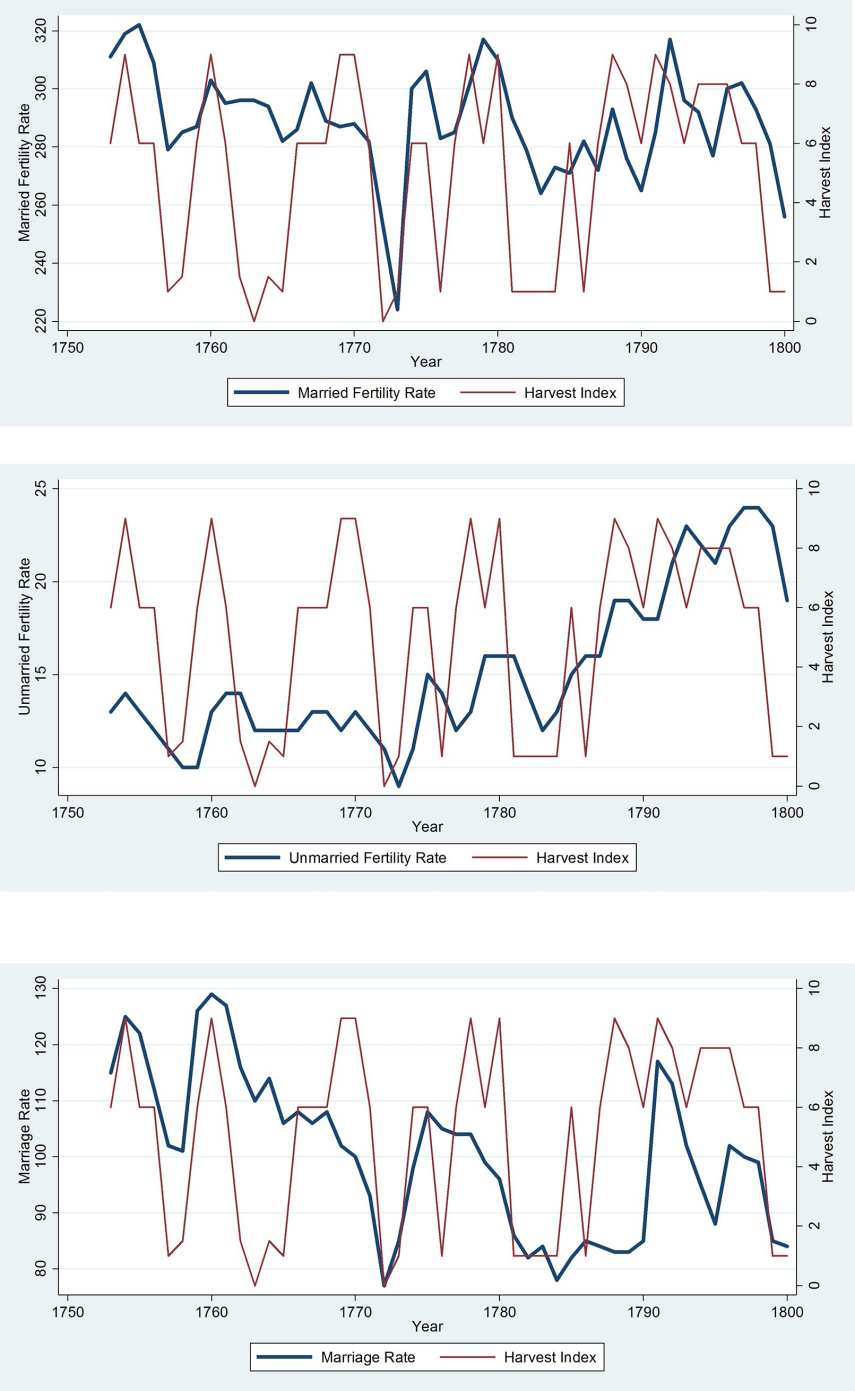
The previous year’s harvest had tremendous impact on 18^th^ century Swedes when it came to. (A) Birthing children in marriage. (B) Birthing children outside of marriage. (C) Deciding whether to marry.

Table 2 provides descriptive statistics and correlation coefficients for the variables used in our analysis. Fig 1 presents bivariate time series graphs of the harvest index relative to married fertility rates, unmarried fertility rates, and marriage rates.

**Table 2:** Summary statistics and correlation coefficients.

Variable	Mean	Std. Dev.	Min	Max	1	2	3	4	5	6	7	8	9	10	11	12	13	14	15	16
1. MFR _t_	287.50	17.86	224.00	322.00	1.00															
2. UFR_t_	15.24	4.19	9.00	24.00	0.19	1.00														
3. MR_t_	99.46	13.79	77.00	129.00	0.61	-0.21	1.00													
4. HARV_t_	4.92	3.12	0.00	9.00	0.46	0.31	0.29	1.00												
5. HARV_t-1_	5.10	3.12	0.00	9.00	0.45	0.47	0.15	0.45	1.00											
6. HARV_t-2_	5.21	3.06	0.00	9.00	-0.02	0.35	-0.07	0.04	0.43	1.00										
7. MB_t_	-0.71	0.34	-1.40	-0.30	0.14	-0.34	0.31	0.04	-0.07	-0.13	1.00									
8. HEIGHT_t_	66.89	0.18	66.50	67.24	0.03	-0.44	0.31	-0.29	-0.16	0.00	0.01	1.00								
9. IDR_t_	204.74	21.98	164.00	286.00	-0.45	-0.32	-0.09	-0.35	-0.08	0.16	0.12	0.13	1.00							
10. POP_t_	2,087.28	137.55	1,866.00	2,352.00	-0.18	0.87	-0.56	0.15	0.19	0.26	-0.47	-0.50	-0.25	1.00						
11. GovCAP_t_	0.11	0.31	0.00	1.00	0.03	-0.24	0.17	0.08	-0.11	-0.27	-0.01	0.37	0.11	-0.23	1.00					
12. GovGIII_t_	0.46	0.50	0.00	1.00	-0.23	-0.06	-0.48	-0.03	-0.11	-0.15	0.26	-0.38	-0.17	0.17	-0.32	1.00				
13. GovDUCK_t_	0.02	0.15	0.00	1.00	0.25	0.21	0.15	0.15	0.19	0.04	0.09	-0.32	-0.08	0.14	-0.05	0.16	1.00			
14. GovGAD_t_	0.11	0.31	0.00	1.00	0.18	0.57	0.01	0.30	0.31	0.25	0.03	-0.42	-0.18	0.44	-0.12	-0.18	0.43	1.00		
15. GovGIV_t_	0.11	0.31	0.00	1.00	-0.02	0.62	-0.14	-0.06	0.08	0.23	-0.71	-0.16	-0.06	0.62	-0.12	-0.32	-0.05	0.10	1.00	
16. WAR_t_	0.24	0.52	0.00	2.00	-0.03	-0.02	0.07	0.15	0.16	-0.07	0.13	0.10	0.13	-0.17	-0.16	0.00	-0.07	-0.16	-0.16	1.00

The results of our base analyses are presented in Table 3. Columns 1–3 present the results of ARMAX regressions where only the key independent variables and autoregressive terms are entered. Columns 4–6 add the covariates, presenting the results of models (1), (2) and (3) described previously.

**Table 3:** The quality of the annual harvest impacted whether people married and had children in 18^th^ century Sweden.

	(1)	(2)	(3)	(4)	(5)	(6)
	Married Fertility Rate	Unmarried Fertility Rate	Marital Rate	Married Fertility Rate	Unmarried Fertility Rate	Marital Rate
***Primary Independent Variables***						
HARV _t_	2.363635*	0.143203*	1.497326**	1.144538	0.136663*	1.457861***
	(1.186020)	(0.071678)	(0.496995)	(0.930445)	(0.063446)	(0.434556)
HARV _t-1_	2.247914**	0.390825***	0.394605	2.287979***	0.359238***	0.616169
	(0.786246)	(0.063545)	(0.553681)	(0.626518)	(0.072522)	(0.548646)
HARV _t-2_	-0.904741	0.143380**	-0.241580	-0.579682	0.178505**	-0.469258
	(0.906514)	(0.054963)	(0.485831)	(0.751089)	(0.061707)	(0.470252)
***Control Variables***						
MB _t_				12.086601	-0.624405	9.051577
				(12.912208)	(1.173372)	(8.754701)
HEIGHT _t_				3.109873	0.780543	5.445357
				(16.518719)	(0.819623)	(8.298233)
IDR _t_				-0.386765***	-0.003373	-0.021669
				(0.086129)	(0.006010)	(0.058814)
POP _t_				-0.068701*	0.020921***	-0.081323*
				(0.034179)	(0.003645)	(0.039552)
GovCAP _t_				-2.024876	-1.043967*	-3.868901
				(12.777510)	(0.456404)	(4.675835)
GovGIII _t_				-4.874154	-0.476661	-6.952372
				(7.086652)	(0.811547)	(7.459953)
GovDUCK _t_				21.745975	-0.163707	7.983146
				(21.043420)	(1.183569)	(20.123697)
GovGAD _t_				-0.430254	0.716602	-2.820262
				(15.365961)	(1.284903)	(14.053593)
GovGIV _t_				23.308712	0.780484	21.587206
				(17.369567)	(1.471816)	(16.850045)
WAR _t_				-3.155492	1.061190	-5.852738
				(3.502798)	(0.561065)	(4.447620)
***N***	**46**	**46**	**46**	**46**	**46**	**46**

* *p* < 0.05

** *p* < 0.01

*** *p* < 0.001

### Hypothesis testing and assessment of effect size

We test the competing predictions of the externality and internality approaches to human population growth. The externality approach provides our null hypothesis (Hypothesis 1) and predicts there to be no relationship between 18th century Sweden’s harvest and subsequent marriage and birthrates, ceteris paribus. The internality approach provides our counter hypothesis (Hypothesis 2) and predicts there to be a positive relationship between 18th century Sweden’s harvest and subsequent marriage and birthrates, ceteris paribus.

#### Married fertility rate

With respect to the married fertility rate, the results in column 4 support Hypothesis 2: prior harvests play an important role in predicting future fertility among married women. Specifically, the estimates indicate that the harvest one year prior exhibits a positive and significant relationship with this year’s fertility rate. This should not be surprising given the lagged nature of fertility; i.e. if childbirth is experienced in a given year (the current year), conception decisions would have most likely been made during the prior year (nine months prior), and we should expect those decisions to have been influenced heavily by the harvest experienced at the time (the calendar year before the year of birth).

In practical terms, the column 4 estimate on HARV_t-1_ shows that relative to a year of famine (HARV = 0), a year of abundance (HARV = 9) leads to approximately 21 additional births per thousand married women one year later. Relative to a mean level of married fertility (288 per 1,000), this constitutes an increase of more than *7 percent* in the married fertility rate. Of note, the column 4 estimates for the current year and two years prior are not statistically significant at conventional levels.

### Unmarried fertility rate

With respect to the unmarried fertility rate, the results in column 5 also confirm that prior harvests play an important role in predicting future fertility among unmarried women. Specifically, the estimates indicate that harvests in all three years, including the current year, play a role in current fertility rates, with the harvest one year prior again appearing to be most important. Of note, the estimated coefficient for a year prior is significantly greater than the estimates for both the current year (p < 0.01) and two years prior (p < 0.01). Again, this should not be surprising given the lagged nature of fertility.

In practical terms the largest estimate on the harvest from one year prior shows that relative to a year of famine (HARV = 0), a year of abundance (HARV = 9) leads to a little more than 3 additional births per thousand (among unmarried women) one year later. Relative to a mean level of unmarried fertility (15 per 1,000), this constitutes an increase of more than *21 percent* in the unmarried fertility rate. Collectively, the three harvest estimates show that relative to three straight years of famine (HARV = 0), three straight years of abundance (HARV = 9) would lead to a little more than 6 additional births per thousand in the current year, an increase of more than *40 percent*.

#### Marriage rates

Finally, with respect to marriage rates, the results in column 6 confirm that harvests again play an important role in predicting decisions to enter marriage. Specifically, the estimates indicate that harvests in the current year play a role in current marriage rates. Of note, the estimates for harvests from one and two years prior are not statistically significant at conventional levels. This pattern may not be surprising, as it implies that the same kind of lags observed affecting fertility were likely not at play in marriage decisions (in contrast to modern western trends toward long engagements).

In practical terms the model estimates that relative to a year of famine (HARV = 0), a year of abundance (HARV = 9) lead to a little more than 13 additional marriages per thousand in that same year. Relative to a mean marriage rate (100 per 1,000), this constitutes an increase of a little more than 13 *percent* in the marriage rate.

Collectively, the results of our models confirm the raw relationships observed in Fig 1 and support Hypothesis 2: the predictions based on the assumptions of the internality approach to human nature and the relative costs of child-bearing.

### Robustness checks

To assess the stability of our findings, we undertook many robustness checks. First, we recognize that any endogeneity in our models could bias our estimates. Perhaps most concerning, in this case, is the potential for reverse causality. Specifically, it may be that our estimates are simply picking up a correlation driven by a relationship that runs in the opposite direction (i.e., marriage rates, and fertility rates drive harvests). Fortunately, the longitudinal nature of our data permits us to explore this possibility. Accordingly, we ran three additional regression models, one for each of the rates used as dependent variables in our main models. Each of these models was specified with the current year’s harvest index as the dependent variable and two years of lagged MFR, UFR and MR, respectively, as the key independent variables. All the covariates from the main models were included, and we included one autoregressive lag of the harvest index in each model, in accordance with the results of a Dickey-Fuller test of HARV_t_. These models allow us to investigate the extent to which prior fertility and marriage rates positively impact future harvests (which could result in positive inter-temporal correlations between the rates and the harvest index in our main models). The results are presented in columns (1) through (3) of Table 4. The results show that prior fertility and marriage rates do not exhibit a significant positive relationship with future harvests. Only one of the estimated coefficients is significant at conventional levels (p < 0.05), but the estimate is negative, consistent with the idea that, if anything, the relationship would work against our main findings. While not completely mitigating the potential for reverse causality, these results give us significant comfort that our main results are not biased by endogeneity.

**Table 4:** Robustness checks show that prior fertility and marriage rates did not exhibit positive relationships with the bounty of future annual harvests in 18^th^ century Sweden.

	(1)	(2)	(3)
	Harvest Quality	Harvest Quality	Harvest Quality
RATE_t-1_	0.007551	-0.102782	0.010669
	(0.026967)	(0.345605)	(0.082516)
RATE_t-2_	-0.052091*	-0.159231	-0.014409
	(0.025174)	(0.337395)	(0.077476)
MB _t_	0.451267	0.870975	0.471043
	(2.025284)	(2.099935)	(1.986048)
HEIGHT _t_	-4.008905	-4.655615	-4.745803
	(3.220811)	(3.764037)	(3.448065)
IDR_t_	-0.051883**	-0.052581*	-0.052565
	(0.019737)	(0.025782)	(0.026964)
POP_t_	0.001222	0.008639	0.001932
	(0.007297)	(0.010030)	(0.009244)
GovCAP_t_	1.417627	1.241699	1.621264
	(1.529106)	(1.700654)	(1.730696)
GovGIII_t_	-1.748856	-1.983912	-1.413521
	(1.673585)	(1.768012)	(1.878425)
GovDUCK_t_	-0.696184	0.215626	-0.366008
	(4.517332)	(6.639709)	(9.069323)
GovGAD_t_	0.953882	0.626453	0.777157
	(2.991955)	(3.230288)	(3.416753)
GovGIV_t_	-1.374405	-1.135319	-1.584722
	(3.060154)	(3.458749)	(3.542139)
WAR_t_	1.181081	1.177557	1.213483
	(0.940486)	(1.016120)	(1.057126)
**RATE**	**Married Fertility Rate**	**Unmarried Fertility Rate**	**Marital** **Rate**
***N***	**46**	**46**	**46**

* *p* < 0.05

** *p* < 0.01

*** *p* < 0.001

Second, our models do not control for annual weather patterns. While we expect weather to be related to harvest quality, it could also be that weather patterns influenced sexual relations and subsequent fertility rates–as it has been found that UK males’ attraction to women becomes higher in colder seasons than warmer seasons [76]. We tested this possibility by examining a series of models in which weather patterns were included as controls. Specifically, we included two measures of weather patterns during the typical growing season from May through October: (i) average temperature and (ii) the number of rainy or snowy days. These measures were obtained from the Bolin Centre for Climate Research at Stockholm University. These data are publicly available and include daily weather observations taken in the city of Stockholm between 1756 and 2013 (Data retrieved from http://bolin.su.se/data/stockholm/ on November 28, 2016). We aggregated these daily weather observations to produce annual growing season measures for temperature and rain/snow between 1756 and 1800. The results of the models including these variables as controls are presented in Table 5, columns (1) through (3). The inclusion of these measures does not have a meaningful impact on our results. The only statistically significant effect is a negative relationship between daily precipitation and unmarried fertility rates. These statistically significant results for daily precipitation and fertility are counter those suggested by the Pawlowski and Sorokowski [76] paper because the rainiest seasons in Sweden are in the warmer months of June through September (https://www.climatestotravel.com/climate/sweden).

**Table 5:** Robustness checks show that the inclusion of weather patterns does not have a discernible impact on the relationship between harvest quality and fertility and marriage rates in 18^th^ century Sweden.

	(1)	(2)	(3)
	Married Fertility Rate	Unmarried Fertility Rate	Marital Rate
***Primary Independent Variables***			
HARV _t_	1.361705	-0.010903	1.267483*
	(0.939020)	(0.084653)	(0.544441)
HARV _t-1_	2.544243**	0.271338***	0.390929
	(0.790841)	(0.081906)	(0.631794)
HARV _t-2_	-0.615828	0.057962	-0.369981
	(0.799704)	(0.056581)	(0.470493)
***Control Variables***			
TEMPERATURE _t_	-1.158074	0.007534	1.203468
	(2.029381)	(0.135762)	(1.239784)
DAYSPRECIPITATION _t_	-0.320367	-0.062937***	0.041741
	(0.255976)	(0.012108)	(0.128879)
MB _t_	10.843380	3.853481***	7.706832
	(14.847760)	(0.338019)	(11.190127)
HEIGHT _t_	11.286218	3.905400*	4.469704
	(16.214364)	(1.619597)	(9.125955)
IDR _t_	-0.374512***	-0.039990***	-0.034455
	(0.096736)	(0.007518)	(0.067974)
POP _t_	-0.076707	0.021928***	-0.079400*
	(0.040435)	(0.001593)	(0.039897)
GovCAP _t_	-3.257456	-0.117841	-2.391837
	(11.458008)	(0.523371)	(4.401277)
GovGIII _t_	-2.164224	-0.773306	-8.131977
	(7.792857)	(0.507262)	(8.628993)
GovDUCK _t_	23.082864	3.605996**	9.962969
	(28.062535)	(1.212658)	(23.647503)
GovGAD _t_	-0.871171	1.053944	-4.492540
	(15.320275)	(0.622323)	(17.062377)
GovGIV _t_	24.512595	4.997527***	20.122383
	(20.015156)	(0.996335)	(22.492230)
WAR _t_	-6.554878	0.859202***	-5.226257
	(5.540143)	(0.255309)	(3.441532)
*N*	45	45	45

* *p* < 0.05

** *p* < 0.01

*** *p* < 0.001

NOTE: These regressions include only 45 observations because the weather data was only available beginning in 1756.

Third, with respect to our control variables, it is possible that temporal lags may impact their relationships with our key independent variables and dependent variables. Therefore, we reran our analysis using the models in Eqs (1), (2) and (3), replacing our control variables with one-year lags of each. We do not report the results here, but they are nearly identical to the estimates presented in Table 3, suggesting that our results are robust to the temporal positioning of our covariates.

Fourth, with respect to our dependent variable, the distribution of the Harvest Index for our observation window resembled a sort of bimodal distribution. Thus, we recoded the Harvest Index (including the lagged versions) as a binomial variable where 0 is equivalent to famine (i.e., HARV < 4) and 1 is equivalent to abundance (i.e., HARV > 4). We then reanalyzed the data using the models in (1), (2) and (3), replacing our continuous versions of the harvest index with these binomial versions. The results are consistent with the estimates presented in columns (4) through (6) of Table 3, suggesting that our results are robust to the coding of the harvest index.

Fifth, as noted in the analysis above, it is possible that the autoregressive lags employed for each of our dependent variables could impact our findings. Although extant literature suggests following the MAIC procedure produces the optimal autoregressive lags, we also assessed autoregressive lags using the NG-Perron Sequential t and SIC procedures. The NG-Perron suggested zero lags was optimal for MFR, two lags were optimal for UFR (same as the MAIC), and five lags were optimal for MR. The SIC suggested one lag was optimal for MFR, two lags were optimal for UFR (same as the MAIC), and one lag was optimal for MR (same as the MAIC). Given that a few of these results differed from the MAIC results, to test the robustness of our findings to the choice of method for identifying the optimal number of lags, we re-ran two MFR models with zero and one lag, respectively, and one MR model with five lags. Again, the results were nearly identical to the results presented in Table 3, leaving our findings unchanged. Thus, our findings are robust to the choice of method for identifying the optimal autoregressive lags.

Lastly, the field of human reproduction ecology suggests an alternative explanation for the harvest’s effect on fertility rates: famine produces malnourished females who are less likely to ovulate, thereby making fertility less likely [77]. Therefore, the human reproduction ecology literature could maintain that the effects of harvest on fertility rates is fully mediated by human physiology rather than human decision making [78]. One way to test this alternative explanation is to examine whether variables reflecting human decision-making mediate the relationship between the harvest and fertility. Fortunately, our data includes information on an important decision people make: the decision to marry. Should the harvest impact birthing children indirectly through marriage, then this would be consistent with the idea that human decision making was at least one factor.

We test for the extent of mediation using Baron and Kenney’s [79] established approach as well as Preacher and Hayes’ [80] bootstrap estimation approach for robustness. Specifically, we hypothesize that last year’s harvest would have a contemporaneous effect on last year’s marriage rate, in turn, impacting this year’s fertility rate. Based on the Baron and Kenny [79] approach, our results are consistent with the theory that last year’s marriage rate completely mediates the relationship between last year’s harvest and this year’s fertility.

Specifically, with respect to the married fertility rate, the results of the first two steps in the Baron and Kenny [79] approach can be seen in Table 3, columns 4 and 6 where we see that the relationship between last year’s harvest and this year’s fertility rate is positive and significant (β = 2.288, p < 0.001) (Step 1), and the relationship between contemporaneous harvest rates and marriage rates is also positive and significant (β = 1.458, p < 0.001) (Step 2). Steps 3 and 4 were assessed as follows, in a regression equating last year’s marriage rate to this year’s fertility rate (controlling for last year’s harvest), we found that marriage rates were positively and significantly related to fertility rates (β = 0.862, p < 0.05) (Step 3) and that the estimated relationship between last year’s harvest and this year’s fertility dropped substantially (from β = 2.288 to β = 1.115) and became insignificant at conventional levels (p = 0.245).

Similarly, based on the Preacher and Hayes [80] approach, we also find evidence of complete mediation based on a bootstrapped test of the overall indirect effect. The Preacher and Hayes [80] method adds a more stringent test of the overall indirect effect based on bootstrapping the estimated indirect effect and its standard error and constructing a 95% confidence interval therefrom. We ran multiple iterations of this method, with each producing consistent, albeit slightly different point estimates for the confidence intervals, with the lower bound oscillating between just below zero (e.g., -0.009) to just above it (e.g., 0.011). The upper bounds were consistently in the 1.75 range. Thus, we feel comfortable that, using the most stringent test, the indirect effect remains significant at the 95% level of confidence. These results are consistent with the idea that the relationships we observe in our main analysis are, in part, a function of endogenous choices made by those living in the 18^th^ century Swedish *bytvång*.

## Discussion and conclusion

Our study was motivated by a long-standing debate surrounding the grand challenge of human population growth. The debate of population growth revolves around two questions: the first is concerning the nature of the social trap and the second concerning its consequences. We aimed at addressing the first question: is population growth a collective trap (*Hypothesis 1*) requiring centralized, bureaucratic intervention, or is it an individual trap (*Hypothesis 2)* conducive to self-organizing? Answering this question not only resolves competing theories about the nature of human population growth, but it also provides leaders and decision-makers with some insight into how to tackle the grand challenge.

When it came to working fields, managing forests, and tending herds, the historical narrative gives evidence that 18^th^ century Swedes unquestionably grappled with a collective trap: villagers slacked chores and daily farm work, leaving the collective vulnerable to the Tragedy of the Commons. Yet, when it came to marriage and child-bearing (whether in or outside of wedlock), our results are consistent with the position that these impoverished, uneducated, commune-living villagers engaged in some level of self-regulating behavior; expanding or contracting marriage and child-bearing based on fluctuations in the annual harvest. Thus, decisions around marriage and child-bearing represent an individual trap and, for society more broadly, the internality approach to population growth may be optimal. Our empirical findings along these lines contribute to our understanding of self-control in social dilemmas, population growth, cooperative behavior in organizations in several ways.

### Hyperbolic discounting in social dilemmas

There is a growing body of work examining the idea of hyperbolic discounting and its effect on self-control in social dilemmas [81, 82]. *Hyperbolic discounting* is where an individual is unable to sacrifice self-interests in the short run and places little weight on long-run objectives [83]. Van Lange et al.’s [84] recent survey of the social dilemma literature calls for “studies that address the time dimension more fully–for example, by using longitudinal research designs … [that] allow us to capture topics such as self-control [and] delay of gratification … in the social dilemma context (p. 145).

The current research meets Van Lange et al.’s [84] request. However, in the context of population growth, we find evidence consistent with the idea that people–even those who are placed in a social dilemma with strong incentives to stress the commons–are quite capable of exercising self-control and resist hyperbolic discounting. The evidence is inconsistent with the externality approach [7] but consistent with the internality approach [23]: the costs internalized by the family for child rearing are greater than those externalized to the community.

### Self-organizing to achieve collective action is possible in large groups

Our findings follow a line of work on collective action in the political economy. The economics Nobel Laureate Elinor Ostrom [6], for example, observed that humans, in small remote groups, can self-organize and self-govern shared resources without a centralized authority. Ostrom’s idea was that people who share resources can avert collective traps through “covenants without a sword” [85]. The metaphors of covenants and swords are like unto Scott’s [86] idea of *institutions*, which: “attends to the deeper and more resilient aspects of social structure. It considers the processes by which structures, including schemas, rules, norms, and routines, become established as authoritative guidelines for social behavior” (p. 460).

Institutions exert their influence on individual and collective behavior through at least three types of pressure: normative, mimetic, and coercive. Ostrom’s metaphor of the covenant is like the informal agreements underlying normative pressures that encourage compliance through self-regulating and self-organizing behaviors. Her metaphor of the sword is like unto the threat of sanctions underlying coercive pressures that derive from formal institutions enforcing behavioral expectations. Ostrom’s research, therefore, suggests that small groups of people can produce individual action that is collectively beneficial through normative pressures alone, without the need for coercion by a central institutional authority.

While proponents of the externality approach have maintained that such self-organizing does not apply to larger groups, e.g. self-organizing order starts to disintegrate with a group size of around 100 [87], our country-level study suggests that Ostrom’s insights may extend to larger societies; e.g. 18^th^ century Sweden. While the self-regulating patterns we observe in our empirical analysis may be an aggregation of local effects, the average size of the relevant locality, i.e. the parish–the center of religious, social and economic life–was nearly 690 members, far more than the maximum group size postulated by the externality approach. Author calculations are based on data retrieved from *Historical Statistics of Sweden–Part 1*. *Population*, *Central Bureau of Statistics*, *Stockholm* [88]. These data provide information on total population of Sweden in 1750 (1,780,678), right at the beginning of our sample period. The earliest data available on the number of parishes is 1866, when there were 2,592. The number of parishes appears to have been relatively stable over time, with the total number declining by only 30 between 1866 and 1967 and therefore we rely on the 1866 figure for our estimate (1,780,678 / 2592 = 687).

### Filling the confusion gap on human population growth

Science was envisioned by the late philosopher Charles Peirce [89] as the mechanism whereby “the ultimate conclusion of every man [about a phenomenon] shall be the same” (p. 18). Our research about human population growth was motivated by observing different conclusions over the grand challenge and how human behavior organizes. The confusion stems from theories resting on competing assumptions about human nature, cognitive capacity, and the nature of the dilemma’s human’s face; e.g. child-bearing as an individual versus collective trap. These assumptions have profound implications for both the study and practice of management.

Indeed, the assumptions underlying the externality (collective trap) and internality (individual trap) perspectives find direct corollaries in the assumptions underlying McGregor’s [41] classic thesis on theory X and theory Y. Consistent with Theory X, the externality approach assumes that decision making occurs in a relatively self-enclosed system: those making the decisions are cognitively challenged and purely self-interested, limiting their ability to see outside their own wants and how their choices will impact the external environment. Consistent with Theory Y, the internality approach [90] assumes that decision making occurs in a relatively open system: those making decisions have absorptive and creative cognitive capabilities, including foresight not only for themselves but for how their decisions affect those around them. By showing that human beings appear to have self-regulated their marital and child-bearing behavior in response to the quality of the harvest in 18^th^ century Sweden, our findings support the idea that, given the right conditions, the natural human desire and capacity for progress and betterment can lead to self-organizing behaviors that improve the collective order.

Under these more hopeful assumptions about human nature, the task of managing and organizing human behavior looks different than what we observe in modern practice. Indeed, the tenets of scientific management [91] and Theory X assumptions have dominated the teaching and practice of management for over the last century; e.g. the fact that most corporations continue to call their chief accounting officer “controller” is telling [92]. In contrast to this more pessimistic view of human nature, our results are consistent with the position that, under the right conditions–e.g. individual goals aligned with collective goals, the absence of constraining bureaucracy and hierarchy–people are capable of self-regulating in ways that result in collective self-organization [93]. From this optimistic perspective, efforts to organize and manage people may be most effectively directed towards creating the right organizational conditions–e.g., improving goal alignment, reducing unnecessary bureaucracy, flattening out the hierarchy–and giving individuals sufficient freedom to self-organize, rather than attempting to motivate behavior through coercive, punitive means. Rather than seeking to control both means and ends, managers may be better off seeking alignment on the ends, engendering commitment on the part of their people, and then letting the means emerge from social interactions.

Google, Inc., has experienced considerable success taking this management-of-ends approach. Google’s clear vision and values, hiring for alignment, flat hierarchy, minimal bureaucracy and open communication, ensure tight commitment on the part of employees and the freedom to self-organize in ways that enhance idea generation and innovation–despite being a firm of considerable size. The human capacity for self-regulation observed in our empirical analysis is consistent with the position that the self-organization approach to motivating human behavior may be valuable for a wide variety of organizations (be they corporations or simple structure organizations or whole societies) and problems (from technological innovation to human population growth).

### Limitations of current research

The current research is not exempt from limitations. Thorngate’s [94] generalizability-accuracy-simplicity typology for assessing the strengths and weaknesses of a study about social behavior is helpful here. Thorngate [94] maintains that a theory about social behavior is more valuable compared to others when it is generalizable, accurate, and simple. In assessing our theory about social behavior, we find that it is strong in some ways and limited in others. First, consider generalization. While we utilized a field setting with archival data, the current research is limited in generalizability since its focus is 18th century Sweden and we cannot speak to other nations and populations. The current research is an early step toward knowing whether population growth is a social dilemma or an individual one.

Regarding accuracy, the current study’s measures are low in precision in that we do not have individual family demographics (e.g. family size, family status, land ownership), birth dates, medical assessments (e.g. the baby’s birth height and weight), and local crop price levels. Fortunately, our research triangulates previous scholarship by Bengtsson and Dribe [50] on human fertility in 18^th^ century Sweden. The Bengtsson and Dribe [50] study uses data taken from a handful of perishes in southern Sweden and shows that individual families controlled their fertility as a function of local grain prices known nine months earlier. Therefore, in combining our findings with the those from the Bengtsson and Dribe [50] paper, the current research provides a “conceptual extension” of the internality approach to human population growth [95].

Lastly, the current research is high in theoretical simplicity. Our results are consistent with the idea that human population growth is an individual trap where the internal benefits of human reproduction are eclipsed by the internal costs associated with child rearing. Of course, alternative mechanisms could explain why marriage and birth rates rose and fell as a function of previous years’ harvests. One explanation for the harvest-marriage relationship could be that poor harvests negatively affected the frequency of social activities (e.g. men meeting women at village festivals) which subsequently reduced the number of marriages in the future. A second explanation involves birth rates. Early work on social dilemmas identify moral suasion as an explanation for cooperation [3]. It is possible that a person living in 18^th^ century Sweden–when facing a poor harvest–chose to abstain from procreation for the good of the village. This alternative mechanism assumes that the costs of rearing a child are greater for the community than for the individual, and we are unable to measure such costs with the current data. Future scholarship may benefit from assessing human perceptions of the costs and benefits of child bearing and rearing experienced by the family and community.

### Future research: Examining the consequences of human population growth

The second question underlying the human population debate motivating our study concerns what happens to the quality of life as population increases? We provide only a preliminary answer to the question here, fueling future researchers to explore a detailed answer to the question. The externality approach assumes that, like animals, human beings are relatively stagnant when it comes to their capacity for technological progress. Even where the door is open to progress, such progress is assumed to be limited [22]; thus, production and consumption remain relatively constant over time [40]. Moreover, under this view, technological innovations have largely negative externalities [22]. Thus, the externality approach predicts, ceteris paribus, that, as the population of a region increases and technology advances, the quality of life decreases over time; e.g. food, water, and energy shortages will occur [96]; and new technologies will only make consumption easier, e.g., vaccines preventing fatal diseases from curbing population growth.

Our results are consistent with a different view of the human mind as “the ultimate resource” capable of technological progress that increases productive capacity. Indeed, the internality approach assumes that individual human productivity changes over time, eventually surpassing consumption, even in the absence of technological progress [35]. When a child is born, that child primarily consumes resources. Yet, as the person ages, is educated, and enters an occupation, they begin to produce more than they consume. Moreover, the productivity phase of a person’s life (about 50 years) is generally more than twice that of the consumption phase, which is about 20 years [23]. Lastly, technological advancements have positive externalities, predicting, ceteris paribus, that as the population of a region increases and technology advances, the quality of life over time increases.

**Fig 2:**
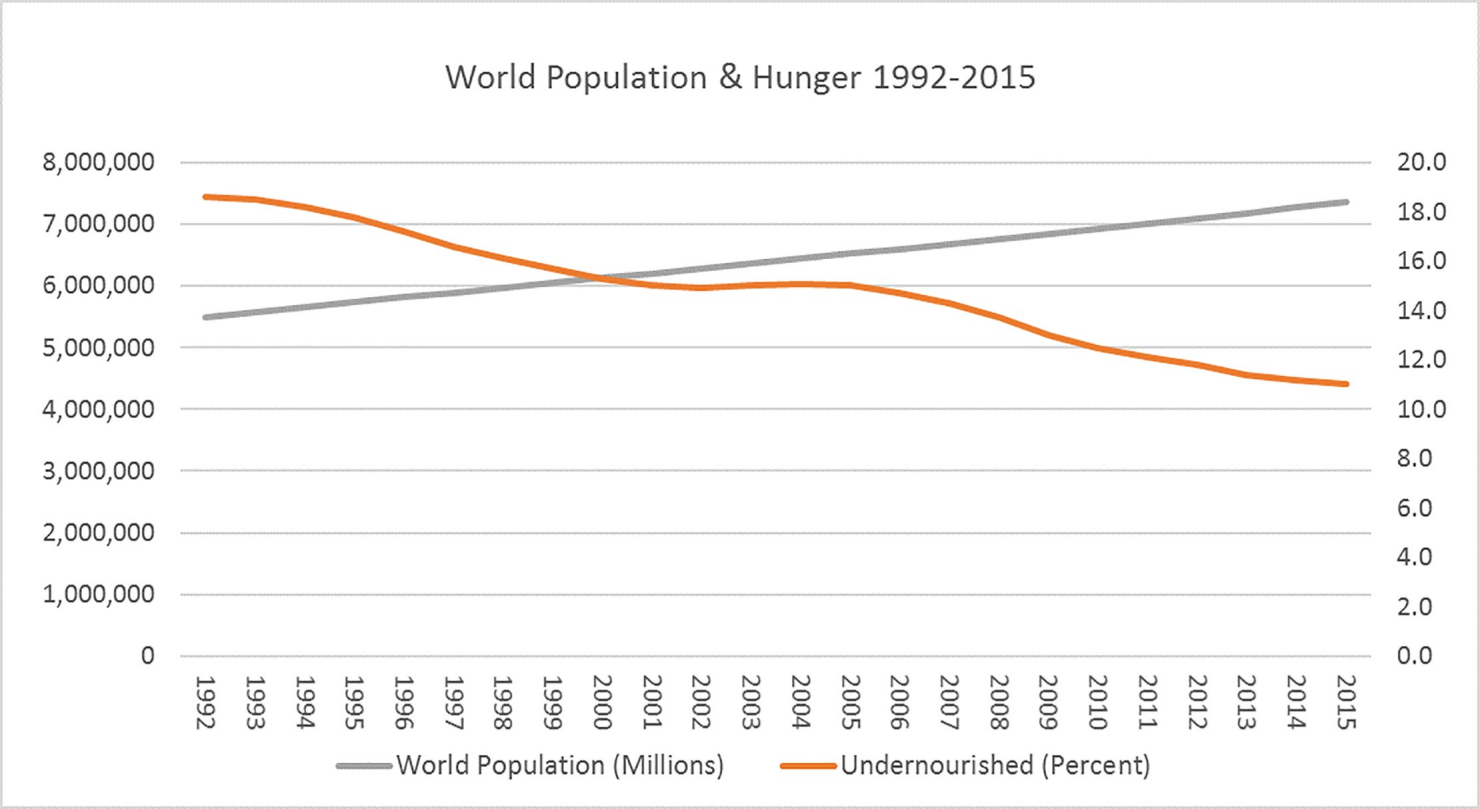
The relationship between world population and world hunger (under-nourishment) 1992 to 2015. Source: Compiled by authors based on data from the FAOStat database maintained by the Food and Agriculture Organization of the United Nations. Data retrieved from http://www.fao.org/faostat/en/#home on January 20, 2017.

To assess the possibility that quality of life improves rather than decays, we conducted a supplemental analysis (see Appendix 1 for details). Using data from the Food and Agriculture Organization of the United Nations, we plotted world population and hunger between 1992 and 2015 (see Fig 2). The trends are consistent with Simon’s [35] view that as the world’s population has risen, the share of Earth’s population that is hungry has declined considerably. To examine whether the implied relationship held under more rigorous econometric analysis, we conducted a more thorough dynamic-panel regression analysis based on a much larger dataset, including the same measures at the country-level for 227 countries world-wide between 1992 and 2015. The results of that analysis suggest that for every doubling of a given country’s population, on average undernourishment falls by 17.27 percent. We estimated our dynamic-panel regression using the Arellano-Bond estimator (xtabond in Stata), an approach that is designed to overcome consistency problems associated with dynamic panel fixed effects estimators. Among other things, this approach relies on a generalized method of moments (GMM) estimator to ensure that the parameters are consistent. Our model included 4 lags of undernourishment and with this level of lags the Arellano-Bond test of serial correlation (estat abond in Stata) suggested that the assumptions underlying the approach were satisfied. A more detailed explanation of the data and methods used in this analysis is available in Appendix 1.

These supplemental findings lend additional credence to the internality approach to the consequences of population growth. More specifically, the results are consistent with the notion that the capacity of the human mind for creativity and innovation means that population growth enhances, rather than detracts from, the quality of human life; e.g., we now have the capacity to feed a much greater share of Earth’s population than was possible just two decades ago. Future scholarship may benefit from further analysis of these data coupled with additional analysis of the impact population growth has on other measures of quality of life; such as innovation and successful treatments of life-threatening diseases. Indeed, Seager’s [97] commentary on population growth suggests that it is not the number of people that determines the fate of city, state, or planet, but how the number of people use the resources. Individuals must address the economic, social, and environmental challenges they face.

Many years ago, Alan Gregg [98] of the Rockefeller Foundation suggested quality of life will only get worse the more mankind is encouraged to grow. Gregg used a metaphor comparing the human population to cancer, a living thing that demands food but never improves the more it is fed. Whether life is getting better on all fronts–in the long run–as population grows is still open for debate, but the current paper does answer the question as to whether humans are like unto cancer. Humans demand food to sustain life, just like cancer, but our findings are consistent with the position that humans can adjust their plans and behavior in response to changing circumstances and resources. The adjustment of plans and behavior can occur without the need for external, centralized bureaucratic interference. Self-organizing behavior is consistent with the internality approach and the idea that population growth may not be a grand challenge, but, in fact, a grand opportunity.

## Appendix 1

### Population Growth and Hunger Across the World, 1992–2015

Using data from the Food and Agriculture Organization (FAO) of the United Nations, we assembled a panel of country-level data representing 227 countries world-wide covering the period between 1992 and 2015. This dataset included information on the population of each country (POP_ct_) and a measure of hunger, the prevalence of undernourishment (UNDER_ct_). We then develop a dynamic panel model as follows:

$$ln({UNDER)}_{ct}=\gamma_{t}+\delta_{c}+{\ln\left(POP\right)}_{ct}+{\ln\left(UNDER\right)}_{ct-1}+v_{ct}$$

Where *γ_t_* represents year effects and *δ_c_* represents country fixed effects, and *v_ct_* represents the error term, which is generally assumed to be uncorrelated over time. Unfortunately, traditional estimation procedures for the model, including ordinary least squares and generalized least squares, result in inconsistent estimates because of endogeneity and Nickel bias.

Fortunately, endogeneity and Nickel bias can be addressed using the Arellano-Bond (AR) approach. The typical approach to addressing endogeneity in regressors is the instrumental variables approach, relying on external instruments. The AR approach is advantageous because it does not require external instruments and instead relies on lagged dependent variables as valid instruments that can be used to derive consistent estimates. It does so, in part, by estimating the model by the generalized method of moments (GMM).

A key question when estimating this kind of model using the AR approach is how many lags to include. The AR approach necessitates a test of whether first differenced errors of a higher order than the first order (i.e., second order) exhibit serial correlation (H_o_: no serial correlation). We find that with four lags of the dependent variable, the null hypothesis of no serial correlation in the second order first-differenced errors cannot be rejected (where as it can with only one, two or three lags), suggesting that with four lags the moments conditions are valid. Accordingly, we estimate the following model using the AR GMM approach:

$$ln({UNDER)}_{ct}=\gamma_{t}+\delta_{c}+{\ln\left(POP\right)}_{ct}+{\ln\left(UNDER\right)}_{ct-1}+{\ln\left(UNDER\right)}_{ct-2}+{\ln\left(UNDER\right)}_{ct-3}+{\ln\left(UNDER\right)}_{ct-4}+v_{ct}$$

The results of our analysis suggest that for every doubling of a given country’s population, undernourishment falls by 17.27 percent, on average. As noted in the paper, the results “are consistent with the notion that the capacity of the human mind for creativity and innovation means that population growth enhances, rather than detracts from, the quality of human life; e.g., we now have the capacity to feed a much greater share of Earth’s population than was possible just two decades ago.”

## References

[1] O’Brien DT (2012) Managing the urban commons. Human Nature 23: 467–489. 10.1007/s12110-012-9156-6 23093459

[2] Chen CC, Chen X-P, Meindl JR (1998) How can coooperation be fostered? The cultural effects of individualism-collectivism. Academy of Management Review 23: 285–304.

[3] Dawes RM (1980) Social dilemmas. Annual Review of Psychology 31: 169–193.

[4] Schofield N (1985) Anarchy, altruism and cooperation. Social Choice and Welfare 2: 207–219.

[5] Kollock P (1998) Social dilemmas: The anatomy of cooperation. Annual Review of Sociology 24: 183–214.

[6] Ostrom E (1990) Governing the commons New York, NY: Cambridge University Press.

[7] Hardin G (1968) The tragedy of the commons. Science 162: 1243–1248. 5699198

[8] Chen X-P, Komorita SS (1994) The effects of communication and commitment in a public goods social dilemma. Organizational Behavior & Human Decision Processes 60: 367–386.

[9] Kocher MG, Martinsson P, Myrseth KOR, Wollbrant CE (2017) Strong, bold, and kind: Self-control and cooperation in social dilemmas. Experimental Economics 20: 44–69.

[10] Martinsson P, Myrseth KOR, Wollbrant C (2012) Reconciling pro-social vs. selfish behavior: On the role of self-control. Judgment and Decision Making 7: 304–315.

[11] Archetti M (2009) The volunteer's dilemma and the optimal size of a social group. Journal of Theoretical Biology 261: 475–480. 10.1016/j.jtbi.2009.08.018 19703470

[12] Murnighan JK, Kim JW, Metzger AR (1993) The volunteer dilemma. Administrative Science Quarterly 38: 515–538.

[13] Vanneste S, Van Hiel A, Parisi F, Depoorter B (2006) From “tragedy” to “disaster”: Welfare effects of commons and anticommons dilemmas. International Review of Law and Economics 26: 104–122.

[14] Winn AM, McCarter MW (2018) Who's holding out? An experimental study of the benefits and burdens of eminent domain. Journal of Urban Economics 105: 176–185.

[15] McCarter MW, Budescu DV, Scheffran J (2011) The give-or-take-some dilemma: An empirical investigation of a hybrid social dilemma. Organizational Behavior and Human Decision Processes 116: 83–95.

[16] Budescu DV, McCarter MW (2012) It’s a game of give and take: Modeling behavior in a give-or-take-some social dilemma. Group Processes & Intergroup Relations 15: 649–667.

[17] Brewer MB, Kramer RM (1986) Choice behavior in social dilemmas: Effects of social identity, group size, and decision framing. Journal of Personality and Social Psychology 50: 543–549.

[18] Cardenas JC, Ahn TK, Ostrom E (2004) Communication and co-operation in a common-pool resource dilemma: A field experiment In: Huck S, editor. Advances in understanding strategic behaviour. London, UK: Palgrave pp. 258–286.

[19] Messick DM, Brewer M (1983) Solving social dilemmas In: Wheeler L, & Shaver P., editor. Review of personality and social psychology. Beverly Hills, CA: Sage Publications pp. 11–44.

[20] Shinada M, Yamagishi T (2007) Bringing back Leviathan into social dilemmas In: Biel A, Eek D, Garling T, Gustafson M, editors. New issues and paradigms in research on social dilemmas. New York, NY: Springer pp. 93–123.

[21] Kerr NL (1990) Applied perspectives on social and temporal dilemmas: An introduction. Social Behavior 5: 201–205.

[22] Hardin G (1974) Living on a lifeboat. BioScience 24: 561–568. 11661143

[23] Simon JL (1996) The ultimate resource 2 Princeton, N.J: Princeton University Press.

[24] Barcelo H, Capraro V (2015) Group size effect on cooperation in one-shot social dilemmas. Scientific Reports 5: 7937 10.1038/srep07937 25605124 PMC4300455

[25] Capraro V, Cococcioni G (2016) Rethinking spontaneous giving: Extreme time pressure and ego-depletion favor self-regarding reactions. Scientific Reports 6: 27219 10.1038/srep27219 27251762 PMC4890119

[26] Rand DG, Greene JD, Nowak MA (2012) Spontaneous giving and calculated greed. Nature 489: 427–430. 10.1038/nature11467 22996558

[27] Komorita SS, Parks CD (1996) Social dilemmas Madison, WI: Brown & Benchmark.

[28] Van den Assem MJ, Van Dolder D, Thaler RH (2012) Split or steal? Cooperative behavior when the stakes are large. Management Science 58: 2–20.

[29] Heckscher EF (1954) An economic history of Sweden Cambridge, MA: Harvard University Press.

[30] Thomas DS (1941) Social and economic aspects of Swedish population movements New York, NY: MacMillan Company.

[31] Scott FD (1977) Sweden: The nation's history Minneapolis, MN: University of Minnesota Press.

[32] Magnusson L (2000) An economic history of Sweden London, UK: Routledge.

[33] Liljeström R (1974) A study of abortion in Sweden Stockholm, Sweden: Norstedt and Söner.

[34] Kent N (2008) A concise history of Sweden Cambridge, UK: Cambridge University Press.

[35] Simon JL (1996) Population matters London, UK: Transaction Publishers.

[36] Cross JG, Guyer MJ (1980) Social traps Ann Arbor, MI: University of Michigan Press.

[37] Read D (2001) Intrapersonal dilemmas. Human Relations 54: 1093–1117.

[38] Tertullian (1870 [203 CE]) A treatise on the soul. Holmes P, translator: Ante-Nicene Christian Library.

[39] Malthus TR (2013 [1798]) An essay on the principle of population, as it affects the future improvement of society New York, NY: Cosimo Classics.

[40] Ehrlich PR (1968) The population bomb New York, NY: Ballenstise Books.

[41] McGregor D (1960) The human side of enterprise New York, NY: McGraw-Hill.

[42] Hardin G (1986) Cultural carrying capacity: A biological approach to human problems. BioScience 36: 599–606.

[43] Ehrlich PR, Ehrlich AH (1990) The population explosion New York, NY: Simon & Schuster.

[44] Hardin G (1995) Living within limits New York, NY: Oxford University Press.

[45] Hardin G (1998) The feast of Malthus. Social Contract 8: 181–187.

[46] Leeming D (2005) The Oxford companion to world mythology Oxford, UK: Oxford University Press.

[47] Godwin W (2013 [1793]) An enquiry concerning political justice Oxford, UK: Oxford University Press.

[48] Kirk D (1996) Demographic transition theory. Population Studies 50: 361–387. 10.1080/0032472031000149536 11618374

[49] Kolk M (2011) Deliberate Birth Spacing in Nineteenth Century Northern Sweden. European Journal of Population 27: 337–359.

[50] Bengtsson T, Dribe M (2006) Deliberate control in a natural fertility population: Southern Sweden, 1766–1864. Demography 43: 727–746. 17236544 10.1353/dem.2006.0030

[51] Hayek F (1973) Rules and order Chicago, IL: University of Chicago Press.

[52] Simon JL (1990) Letters to the editor of Science and reply In: Simon JL, editor. Population matters. London, UK: Transaction Publishers pp. 55–62.

[53] Wattenberg B (1982) Is the era of limits running out? A conversation with Garrett Hardin and Julian Simon. Public Opinion 5: 45–56.

[54] Kakade R (2015) Is overpopulation a legitimate threat to humanity and the planet?. New York Times. http://www.nytimes.com/roomfordebate/2015/2006/2008/is-overpopulation-a-legitimate-threat-to-humanity-and-the-planet.

[55] Dempsey J (2015) Fixing biodiversity loss. Environment and Planning A 47: 2555–2572.

[56] Sandberg J, Alvesson M (2011) Ways of constructing research questions: gap-spotting or problematization? Organization 18: 23–44.

[57] Sundin J, Willner S (2007) Social change and health in Sweden Stockholm, Sweden: Swedish National Institute of Public Health.

[58] Stafanson R (1939) Swedish housing and community planning Ann Arbor, MI: University of Michigan.

[59] Isaksson O (1967) Bystämma and bystadga Uppsala, Sweden: Almqvist and Wiksell.

[60] Arbak E, Villeval M (2013) Voluntary leadership: Motivation and influence. Social Choice and Welfare 40: 635–662.

[61] Dawes RM, McTavish J, Shaklee H (1977) Behavior, communication, and assumptions about other people’s behavior in a commons dilemma situation. Journal of Personality and Social Psychology 35: 1–11.

[62] Chakravorti B, Conley JP, Taub B (2002) Probabilistic cheap talk. Social Choice and Welfare 19: 281–294.

[63] Hallendorff C, Schück A (1929) History of Sweden New York, NY: AMS Press.

[64] Westley FR (1990) Middle managers and strategy: Microdynamics of inclusion. Strategic Management Journal 11: 337–351.

[65] McCarter MW, Northcraft GB (2007) Happy together? Insights and implications of viewing managed supply chains as a social dilemma. Journal of Operations Management 25: 498–511.

[66] Mintzberg H (1983) Structure in fives: Designing effective organizations Upper Saddle River, NJ: Prentice-Hall.

[67] Sundin J (1981) Control, punishment and reconciliation: A case study of parish justice in Sweden before 1850 In: Brändström A, Sundin J, editors. Tradition and transition: Studies in microdemography and social change. Umeå, Sweden: Umeå Universitet pp. 9–65.

[68] Olsson M (1998) Ekonomisk-demografiska kortsiktiga samband i Skåne [The economic-demographic short-term relationship in Skåne] 1690–1855 Ekonomisk-historiska institutionen: Lunds universitet.

[69] Lundh C (2003) Swedish marriages, customs, legislation and demography in the eighteenth and nineteenth centuries. Lund Papers in Economic History 88: 1–69.

[70] Sandin B (2013) Infanticide, abortion, children, and childhood in Sweden: 1000–1980 In: Fass PS, editor. Routledge history of childhood in the western world. Didcot, United Kingdom: Taylor and Francis pp. 360–379.

[71] Sandberg LG, Steckel RH (1987) Heights and economic history: The Swedish case. Annals of Human Biology 14: 101–109. 3300516 10.1080/03014468700006842

[72] Cronholm NN (1902) A history of Sweden from the earliest times to the present day: N.N. Cronholm.

[73] Sundbärg G (1913) Emigrationsutredningen: Betänkande Stockholm, Sweden: Kungl. Boktryckeriet. P. A. Norstedt & Söner.

[74] Samuelsson M, Rådestad I, Segesten K (2001) A waste of life: Fathers' experience of losing a child before birth. Birth 28: 124–130. 11380384 10.1046/j.1523-536x.2001.00124.x

[75] Ng S, Perron P (2001) Lag length selection and the construction of unit root tests with good size and power. Econometrica 69: 1519–1554.

[76] Pawlowski B, Sorokowski P (2008) Men's attraction to women's bodies changes seasonally. Perception 37: 1079–1085. 10.1068/p5715 18773730

[77] Lager C, Ellison PT (1990) Effect of moderate weight loss on ovarian function assessed by salivary progesterone measurements. American Journal of Human Biology 2: 303–312. 10.1002/ajhb.1310020312 28520285

[78] Wood JW (2017) Dynamics of human reproduction: Biology, biometry, demography New York, NY: Routledge.

[79] Baron RM, Kenny DA (1986) The moderator-mediator variable distinction in social psychological research: Conceptual, strategic, and statistical considerations. Journal of Personality and Social Psychology 51: 1173–1182. 3806354 10.1037//0022-3514.51.6.1173

[80] Preacher KJ, Hayes AF (2008) Asymptotic and resampling strategies for assessing and comparing indirect effects in multiple mediator models`. Behavior Research Methods 40: 879–891. 18697684 10.3758/brm.40.3.879

[81] Hendrickx L, Poortinga W, van der Kooij R (2001) Temporal factors in resource dilemmas. Acta Psychologica 108: 137–154. 11569759 10.1016/s0001-6918(01)00032-4

[82] Yi R, Buchhalter AR, Gatchalian KM, Bickel WK (2007) The relationship between temporal discounting and the prisoner's dilemma game in intranasal abusers of prescription opioids. Drug and Alcohol Dependence 87: 94–97. 10.1016/j.drugalcdep.2006.07.007 16930862

[83] Rachlin H (2006) Notes on discounting. Journal of the Experimental Analysis of Behavior 85: 425–435. 10.1901/jeab.2006.85-05 16776060 PMC1459845

[84] Van Lange PAM, Balliet D, Parks CD, Van Vugt M (2014) Social dilemmas: The psychology of human cooperation Oxford, UK: Oxford University Press.

[85] Ostrom E, Walker J, Gardner R (1992) Covenants with and without a sword: Self-governance is possible. American Political Science Review 86: 404–417.

[86] Scott WR (2005) Institutional theory In: Smith KG, Hitt MA, editors. Great minds in management: The process of theory development. Oxford, UK: Oxford University Press.

[87] Hardin G (1989) There is no global population problem. The Humanist July/August: 19–22.

[88] Historical Statistics of Sweden (1969) Population: Part 1 Stockholm, Sweden: Central Bureau of Statistics.

[89] Peirce CS (1955) The fixation of belief In: Buchler J, editor. Philosophical writings of Peirce. Mineola, NY: Dover pp. 5–22.

[90] Simon JL (2009) Hoodwinking the nation New Brunswik, NJ: Transaction.

[91] Frederick T (1911) The principles of scientific management New York, NY: Harper Bros.

[92] Stewart M (2006) The management myth. The Atlantic June: https://www.theatlantic.com/magazine/archive/2006/2006/the-management-myth/304883/.

[93] Hayek F (1976) The mirage of social justice Chicago, IL: University of Chicago Press.

[94] Thorngate W (1976) Possible limits on a science of social behavior In: Strickland LH, Aboud FE, Gergen KJ, editors. Social psychology in transition. New York, NY: Springer. pp. 121–139.

[95] Tsang EWK, Kwan K-M (1999) Replication and theory development in organizational science: A critical realist perspective. Academy of Management Review 24: 759–780.

[96] Diamond J (2005) Collapse: How societies choose to fail or succeed New York, NY: Penguin.

[97] Seager J (2000) The 6-billionth baby. Environment and Planning A 32: 1711–1713.

[98] Gregg A (1955) A medical aspect of the population problem. Science 121: 681–682. 14372973 10.1126/science.121.3150.681

